# The role of neurofilament light chain and other fluid biomarkers in cerebellar ataxias

**DOI:** 10.1093/braincomms/fcag313

**Published:** 2026-10-06

**Authors:** Charlotte Mouraux, Magda M Santana, Liana S Rosenthal, Andrea Cortese, Filippo M Santorelli, Puneet Opal, Giulia Coarelli

**Affiliations:** Sorbonne Université, Paris Brain Institute—ICM, Inserm, CNRS, APHP, Pitié-Salpêtrière University Hospital, Paris 75013, France; GIGA—CRC Human Imaging—Rare Movement Disorders Research Group, University of Liège, Liège 4000, Belgium; Department of Human Genetics, University Hospital of Liège, Liège 4000, Belgium; Sorbonne Université, Paris Brain Institute—ICM, Inserm, CNRS, APHP, Pitié-Salpêtrière University Hospital, Paris 75013, France; Center for Neuroscience and Cell Biology, University of Coimbra (CNC-UC), Coimbra 3004-504, Portugal; Center for Innovative Biomedicine and Biotechnology (CIBB), Coimbra 3004-504, Portugal; Gene Therapy Center of Excellence (GeneT), Coimbra 3004-504, Portugal; Department of Neurology, Johns Hopkins University School of Medicine, Baltimore, MD 21287, USA; Department of Neuromuscular Diseases, UCL Queen Square Institute of Neurology, London WC1N 3BG, UK; Department Medical Biotechnology and Translational Medicine, Università Degli Studi Di Milano, Milan 20133, Italy; Unit of Rare Neurological Diseases, Department of Clinical Neurosciences, Fondazione IRCCS Istituto Neurologico Carlo Besta, Milan 20133, Italy; Neurobiology and Molecular Medicine Unit, IRCCS Fondazione Stella Maris, Pisa 56128, Italy; Denning Ataxia Center, Department of Neurology, Northwestern University Feinberg School of Medicine, Chicago, IL 60611, USA; Sorbonne Université, Paris Brain Institute—ICM, Inserm, CNRS, APHP, Pitié-Salpêtrière University Hospital, Paris 75013, France; Genetics Department, DMU BioGeMH, Pitié-Salpêtrière University Hospital, Paris 75013, France

**Keywords:** hereditary cerebellar ataxias, fluid biomarkers, neurofilament light chain, target engagement

## Abstract

The genetic and phenotypic heterogeneity of hereditary ataxias complicates the design of clinical trials since each ataxia progresses at different rates. Consequently, robust biomarkers are urgently needed to monitor therapeutic efficacy and safety of potential therapeutic interventions. Neurofilament light chain has emerged as a leading candidate, serving as a sensitive pharmacodynamic biomarker of neuronal injury and treatment response in neurodegenerative disorders. This review explores the utility of neurofilament light chain across various ataxias, including polyglutamine spinocerebellar ataxias, Friedreich’s ataxia, multiple system atrophy, and other genetic ataxias, such as ataxias associated with *RCF1* and *SPG7* genes. We synthesize evidence from recent studies that have used ultra-sensitive assays. These studies consistently demonstrate elevated neurofilament light chain levels in cerebrospinal fluid and blood, correlating with disease severity, progression and neuroimaging findings. Notably, neurofilament light chain levels rise during the presymptomatic stage in spinocerebellar ataxias, highlighting its potential for stratifying individuals for trial enrolment and tracking therapeutic effects. A treatment-induced reduction in neurofilament light chain could function as a surrogate endpoint for target engagement, analogous to its validated use in amyotrophic lateral sclerosis. Finally, we discuss the results of other studies conducted on other biomarkers, including glial fibrillary acidic protein, total tau and phosphorylated tau, which may reflect complementary aspects of neurodegeneration and glial activation. Glial fibrillary acidic protein may provide insights into astroglial reactivity and neuroinflammation. Similarly, alteration in total tau and phosphorylated tau levels may suggest dysfunction in neuronal cytoskeleton and axonal integrity. The review also focuses on target-engagement biomarkers, including polyglutamine-expanded ataxin proteins in spinocerebellar ataxias and frataxin in Friedreich’s ataxia, as critical tools to complement the ataxia biomarker toolkit. The quantification of mutant ataxin proteins provides a mechanistic readout for emerging gene-silencing or protein-lowering therapies. In parallel, the measurement of frataxin levels serves as a pharmacodynamic marker in therapeutic strategies aiming to restore frataxin expression. These target-engagement biomarkers are valuable in early phases of clinical trials, which mainly aim to demonstrate the biological activity of a treatment before later-phase trials assessing clinical efficacy. Finally, we report on somatic instability in polyglutamine spinocerebellar ataxias as an emerging and highly relevant therapeutic target. CAG repeat mosaicism is gene specific and correlates with disease progression. Integrating measures of repeat instability with neurodegenerative biomarkers could provide a comprehensive monitoring approach in clinical studies. This overview underscores the potential of fluid biomarkers, particularly neurofilament light chain, in accelerating therapeutic development for hereditary ataxias.

## Teaser

Mouraux *et al*. review fluid biomarkers in hereditary cerebellar ataxias, highlighting neurofilament light chain as a sensitive marker of disease activity, progression and treatment response, detectable before symptom onset. They discuss complementary and target-engagement biomarkers. Combining these with digital biomarkers could improve patient stratification, therapeutic monitoring and clinical trial design.

## Graphical Abstract

**Graphical Abstract:**
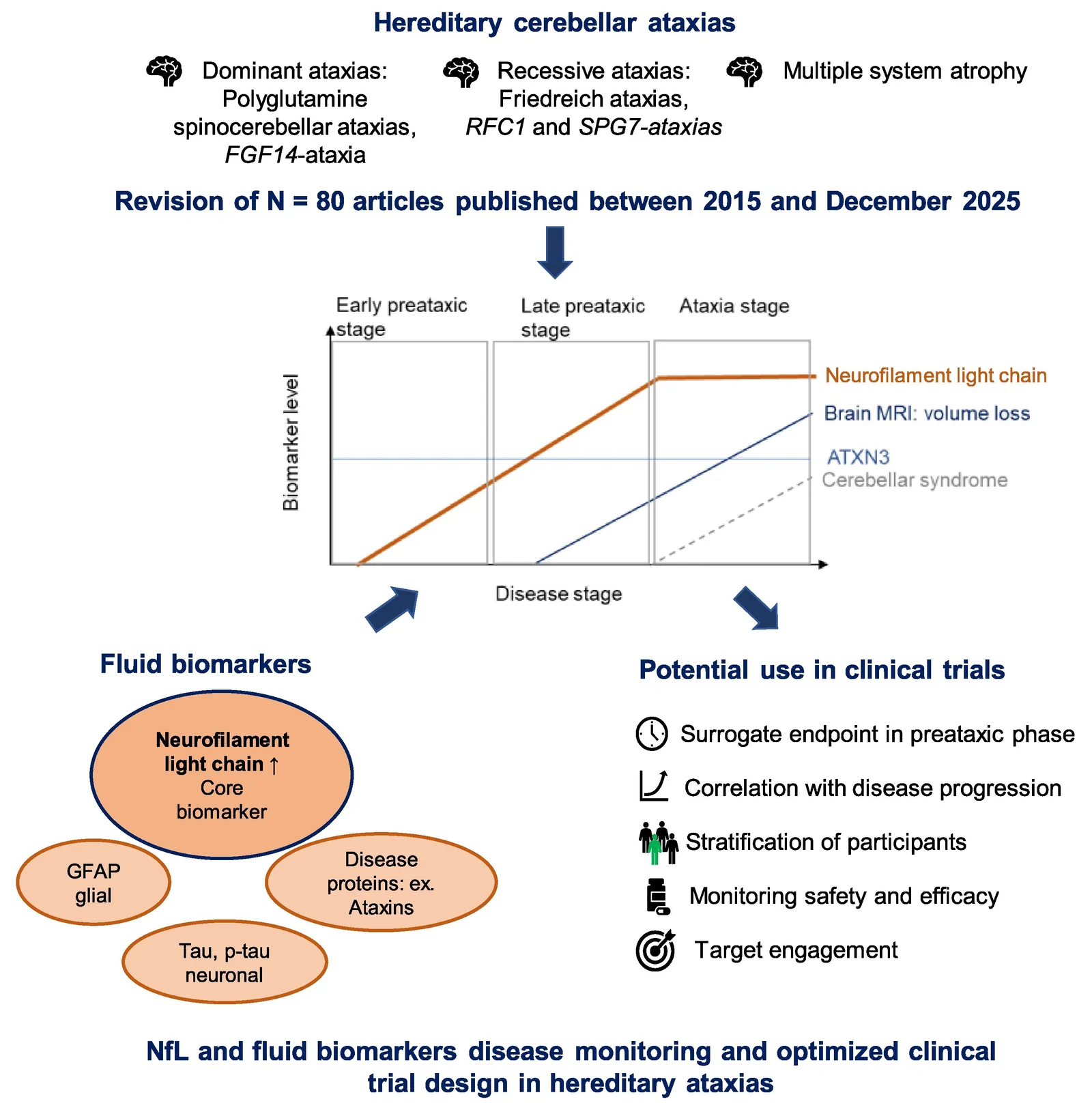

## Introduction

Fluid biomarkers are defined as biological measurements that provide reproducible and quantitative data about specific diseases or disease groups. These markers can be assessed across various human sample types. In neurological disorders, the most frequently analysed samples are CSF, which bathes the central nervous system and more closely reflects neuronal metabolism, and blood, which offers practical accessibility using relatively simple venipuncture. Identifying reliable biomarkers and characterizing the presymptomatic phase are therefore essential for carrier stratification, personalized disease prediction and the design of preventive clinical trials. Among neurodegenerative disease biomarkers, neurofilaments (Nf) have been the most extensively studied. Nf are neuron-specific intermediate filaments composed of three main subunits: light (NfL), medium and heavy chains. They play a critical role in the radial growth of axons and are essential for maintaining axonal diameter, which directly influences the speed and efficiency of neuronal signal transmission. Among these, NfL is a key structural component of the neuronal cytoskeleton. Its concentration in CSF and blood increases in response to axonal damage caused by various pathological processes, including vascular, traumatic, infective and neurodegenerative conditions.^1^

The single-molecule array (Simoa) and Meso Scale Discovery (MSD) assays are antibody-based methods that have enabled ultra-sensitive quantification of NfL, surpassing conventional methods, such as enzyme-linked immunosorbent assay and electrochemiluminescence-based assays in terms of accuracy and detection limits.^2^ Elevated NfL levels are now recognized as a reliable molecular biomarker of neuronal damage and have been consistently reported across a wide ‘spectrum’ of neurodegenerative diseases. There is a well-established correlation between NfL concentrations in CSF and blood, making it a readily accessible biomarker of neurodegenerative processes.^1,3,4^ NfL levels have been shown to correlate with disease stages, clinical severity and neuroimaging outcomes across several neurological disorders, including amyotrophic lateral sclerosis,^5-7^ Alzheimer’s disease,^8-10^ multiple sclerosis,^11,12^ frontotemporal dementia and Huntington’s disease.^13-15^ NfL levels in CSF and blood have also been investigated as biomarkers of neurodegeneration in hereditary ataxias.^16-21^ The correlation between plasma and CSF NfL is strong, regardless of the assay used,^4^ supporting the widespread use of plasma NfL measurement as a less invasive alternative to CSF sampling.

In this review, we explore the potential of NfL as a diagnostic and prognostic biomarker across various hereditary ataxias, including the most common autosomal dominant and recessive forms, and multiple system atrophy (MSA). We also discuss other promising biomarkers, such as glial fibrillary acidic protein (GFAP), total tau (t-tau), phosphorylated tau (p-tau), and disease-specific proteins.

## Materials and methods

We searched PubMed for English-language articles published between 2015 and December 2025. The start date was chosen because the first report describing GFAP in ataxia was published in 2015,^22^ while the first study on NfL in ataxia dates from 2018.^23^ The search terms ‘Neurofilament’, ‘Neurofilament light chain’, ‘GFAP’, ‘tau’, ‘phosphotau’, ‘p-tau’, ‘ATXN3’, ‘frataxin’, ‘hereditary cerebellar ataxia’, ‘spinocerebellar ataxia’, ‘SCA’, ‘polyglutamine diseases’, ‘SCA and biomarkers’, ‘Friedreich’s ataxia’, ‘CANVAS’, ‘RFC1’, ‘SPG7’, ‘FGF14’, ‘SCA27B’, ‘MSA’ and ‘MSA-C’, as well as individual disease-associated genes, were used. For recessive ataxias, only the most frequent forms were included [Friedreich’s ataxia, CANVAS and SPG7]. Inclusion criteria comprised original studies reporting quantitative fluid biomarker measurements (CSF, blood derivatives and urine) in genetically or clinically defined ataxia cohorts. Exclusion criteria included case reports and studies lacking quantitative biomarker data. The study selection process followed a structured screening of titles and abstracts. When the abstract was considered relevant to this review, the authors reviewed the article in detail to confirm inclusion. The final reference list was generated based on the relevance to the topics covered in this review. Summary tables were prepared reporting the main findings of each study for NfL (Table 1; Supplementary Table 1), GFAP (Table 2), tau and p-tau (Table 3) and disease-associated proteins (Table 4).

**Table 1:** Summary of longitudinal studies evaluating NfL levels in polyQ ataxias and Friedreich’s ataxia

Disease	Type of study	Sample	Groups	*N*	Sex (female, %)	Age (years)	SARA score	Assay/platform	NfL (pg/mL)	Main findings	Ref
**SCA1**	Longitudinal	Plasma	PA + A versus CTRL	17 versus 19	NA	45 versus 50	10.7 versus 1.1	Simoa HD-1	25 versus 10	↑ in PA + A Cut-off to A = 16.87 pg/mL No changes after 2 yr	^24^
		Plasma	PA + A versus CTRL	34 versus 20	45 versus 43	51 versus 50	13.0 versus 0	ELLA	35 versus 13	↑ in PA + A No changes after 1 yr	^25^
		Serum	PA versus A versus CTRL	23 versus 17 versus 89	74 versus 47 versus 55	26 versus 45 versus 31	0.5 versus 12.5 versus NA	Simoa HD-X	16 versus 32 versus 6	↑ in A > PA > CTRL ↑ in PA closer to onset No changes after 2.7 yr	^17^
		CSF	PA + A versus CTRL	21 versus 21	45 versus 43	NA	12.5 versus 0	ELLA	3169 versus 739	↑ in PA + A No changes after 1 yr	^25^
**SCA2**	Longitudinal	Plasma	PA + A versus CTRL	13 versus 19	NA	45 versus 50	13.3 versus 1.05	Simoa HD-1	20 versus 10	↑ in PA + A. Cut-off to A = 19.1 pg/mL No changes after 2 yr	^24^
		Plasma	PA + A versus CTRL	5 + 10 versus 10	33 versus 40	41 versus 40	4 versus 0	Simoa HD-1	14 versus 5	↑ in PA + A No changes after 1 yr	^26^
**SCA3**	Longitudinal	Plasma	PA + A versus CTRL	19 versus 19	NA	51 versus 50	14.1 versus 1.05	Simoa HD-1	35 versus 10	↑ in PA + A Cut-off to A = 16.04 pg/mL No changes after 2 yr	^24^
		Plasma	PA versus A versus CTRL	32 versus 179 versus 92	56 versus 50 versus 60	34 versus 52 versus 45	1.0 and 13.0 versus 0	Simoa HD-X	17 versus 27 versus 7	↑ 20 years before onset	^27^
**SCA7**	Longitudinal	Plasma	PA + A versus CTRL	13 versus 19	NA	46 versus 50	10.2 versus 1.05	Simoa HD-1	26 versus 10	↑ in PA + A Cut-off to A = 16.67 pg/mL No changes after 2 yr	^24^
		Plasma	PA + A versus CTRL	9 + 6 versus 10	53 versus 40	38 versus 40	2 versus 0	Simoa HD-1	16 versus 5	↑ in PA + A No changes after 1 yr	^26^
**Friedreich’s ataxia**	Longitudinal	Plasma	A versus CTRL	12 versus 12	50 versus 50	15 versus 15	16.4 versus NA	ELLA	29 versus 5	↑ in A No changes after 1 yr	^28^
		Plasma	A versus CTRL	50 versus 18	48 versus 50	28 versus 26	20.8 versus NA	ELLA	22 versus 6	↑ in A No changes after 1 yr	^28^
		Plasma	A + PA versus CTRL	187 versus 127	53 versus 53	31 versus NA	NA	Simoa HD-1	NA	↑ in A + PA ↓ with time in <35 yr old	^29^
		Serum	PA versus A versus CTRL	13 versus 85 versus 19	46 versus 49 versus 47	38 versus 31 versus 36	NA	Simoa HD-1	8 versus 18 versus 8	↑ in A > PA ≈ CTRL No changes after 1 yr	^30^
		Serum	A versus CTRL	99 versus 30	NA	38 versus 45	NA	Simoa HD-1	26 versus 21	↑ in A No changes after 2 yr	^31^

Data are presented as mean or median. Values were rounded to the nearest integer (age, sex and NfL) or to one decimal place (SARA score). For longitudinal studies, the data presented correspond to baseline. Demographic and clinical data (age, sex and SARA) are reported as provided in each manuscript; due to missing data, in many studies, the number of individuals characterized for age and/or SARA does not always correspond to the number of individuals included in the NfL analysis.

A, ataxic; CTRL, control; NA, not available; PA, preataxic; A, ataxic: Scale for the Assessment and Rating of Ataxia; yr, year/s.

**Table 2:** Summary of studies evaluating GFAP levels in hereditary cerebellar ataxias and MSA-C

Disease	Type of study	Sample	Groups	*N*	Sex (female, %)	Age (years)	SARA score	Assay/platform	GFAP (pg/mL)	Main findings	Ref
**SCA1**	Cross-sectional	CSF	A versus CTRL	5 versus 5	20 versus 60	52 versus 51	11.1 versus 0.375	ELISA	360 versus 290	No differences	^32^
	Longitudinal	Plasma	PA + A versus CTRL	34 versus 20	45 versus 43	51 versus 50	13.0 versus 0	Simoa SR-X	125.8 versus 106.8	No differences ↑ with age No changes after 1 yr	^25^
		CSF	PA + A versus CTRL	21 versus 21	45 versus 43	NA	12.5 versus 0	ELISA	1800 versus 690	↑ in PA + A No changes after 1 yr	^25^
**SCA2**	Cross-sectional	CSF	A versus CTRL	6 versus 5	17 versus 60	54 versus 51	9.7 versus 0.375	ELISA	680 versus 290	↑ in A	^32^
**SCA3**	Cross-sectional	Plasma	PA versus A versus CTRL	23 versus 120 versus 172	57 versus 58 versus 53	35 versus 52 versus 49	1.0 versus 13.5 versus NA	Simoa HD-1	NA	↓ in PA and A < CTRL	^33^
		Serum	A versus CTRL	136 versus 155	25 versus 21	38 versus 37	10.65 versus NA	ELISA	8860 versus 3930	↑ in A	^22^
**SCA6**	Cross-sectional	CSF	A versus CTRL	5 versus 5	60 versus 60	53 versus 51	15.9 versus 0.375	ELISA	310 versus 290	No differences	^32^
**SCA7**	Cross-sectional	Plasma	PA versus A versus CTRL	3 versus 10 versus 8	67 versus 50 versus 63	38 versus 40 versus 48	0 versus 0 versus 11.5	Simoa HD-1	62 versus 83 versus 48	No differences	^34^
		CSF	PA versus A versus CTRL	3 versus 7 versus 8	67 versus NA versus 63	38 versus 40 versus 48	0 versus 0 versus 11.5	Simoa HD-1	14 881 versus 9318 versus 8906	No differences	^34^
**SCAs^a^/MSA-C**	Cross-sectional	Plasma	SCAs versus MSA-C versus CTRL	60 versus 56 versus 74	50 versus 39 versus 55	50 versus 57 versus 55	10.6 versus 11.3 versus NA	Simoa HD-X	69 versus 95 versus 59	↑ in MSA-C versus CTRL ↓ in SCAs versus MSA-C SCAs ≈ CTRL	^35^
**Friedreich’s ataxia**	Cross-sectional	Plasma	A versus CTRL	33 versus 13	61 versus 54	34 versus 37	20.4 versus NA	Simoa HD-1	99 versus 60	↑ in A	^36^
	Longitudinal	Plasma	PA + A versus CTRL	187 versus 127	53 versus 53	31 versus NA	NA	Simoa HD-1	NA	↑ in PA + As (slight increase)	^29^
**MSA-C**	Cross-sectional	Plasma	MSA-C versus SCAs versus CTRL	58 versus 29 versus 100	43 versus 45 versus 56	59 versus 47 versus 60	NA	Simoa HD-X	98 versus 66 versus 69	↑ in MSA-C > SCAs ≈ CTRL	^37^
		CSF	A versus CTRL	5 versus 5	60 versus 60	55 versus 51	20.7 versus 0.375	ELISA	710 versus 290	↑ in A	^32^

Data are presented as mean or median. Values were rounded to the nearest integer (age, sex and GFAP) or to one decimal place (SARA score). For longitudinal studies, the data presented correspond to baseline. Demographic and clinical data (age, sex and SARA) are reported as provided in each manuscript; due to missing data, in many studies, the number of individuals characterized for age and/or SARA does not always correspond to the number of individuals included in the GFAP analysis.

A, ataxic; CTRL, control; MSA-C, multiple system atrophy—cerebellar phenotype; NA, not available; PA, preataxic; SARA, Scale for the Assessment and Rating of Ataxia; yr, year/s.

^a^SCA1 (*n* = 2), SCA2 (*n* = 7), SCA3 (*n* = 27), SCA6 (*n* = 1), SCA7 (*n* = 1), SCA8 (*n* = 4), SCA36 (*n* = 1), other SCAs (*n* = 17).

**Table 3:** Summary of studies evaluating t-tau and p-tau levels in hereditary cerebellar ataxias and MSA-C

Disease	Type of study	Sample	Groups	*N*	Sex (female, %)	Age (years)	SARA score	Assay/platform	t-tau (pg/mL)	p-tau (pg/mL)	Main findings	Ref
**SCA1**	Longitudinal	CSF	PA + A versus CTRL	21 versus 21	45 versus 43	NA	12.5 versus 0	ELISA	289 versus 323	32.0 versus 32.4	No differences	^25^
**SCA2**	Cross-sectional	CSF	A versus CTRL	6 versus 5	17 versus 60	54 versus 51	9.7 versus 0.4	ELISA	109.7 versus ∼43.7	NA	↑ in A	^32^
	Longitudinal	Plasma	PA + A versus CTRL	5 + 10 versus 10	33 versus 40	41 versus 40	4 versus 0	Simoa HD-1	5.9 versus 6.5	1.7 versus 1.5	No differences No changes after 1 yr	^26^
		CSF	PA + A versus CTRL	15 versus 8	33 versus 40	41 versus 40	4 versus 0	Simoa HD-1	128 versus 116	26.7 versus 27.0	No differences No changes after 1 yr	^26^
**SCA3**	Cross-sectional	Plasma	PA versus ataxic versus CTRL	23 versus 120 versus 172	57 versus 58 versus 53	35 versus 52 versus 49	1.0 versus 13.5 versus NA	Simoa HD-1	2159 versus 1667 versus 1447	NA	↑ in PA and young A	^33^
		CSF	PA versus ataxic	8 versus 3	NA	NA	NA	Simoa HD-1	384 versus 227 versus NA	51.0 versus 32.5 versus NA	↑ t-tau and p-tau in PA > A	^33^
**SCA6**	Cross-sectional	CSF	A versus CTRL	5 versus 5	60 versus 60	53 versus 51	15.9 versus 0.4	ELISA	52.2 versus 43.7	NA	No differences	^32^
**SCA7**	Longitudinal	Plasma	PA + A versus CTRL	5 + 10 versus 10	53 versus 40	38 versus 40	2 versus 0	Simoa HD-1	6.8 versus 6.5	1.4 versus 1.7	No differences No changes after 1 yr	^26^
		CSF	PA + A versus CTRL	15 versus 14 versus 8	53 versus 40	38 versus 40	2 versus 0	Simoa HD-1	126 versus 116	30.8 versus 27.3	No differences No changes after 1 yr	^26^
**SCAs^a^/MSA-C**	Cross-sectional	Plasma	SCAs versus MSA-C versus CTRL	60 versus 56 versus 74	50 versus 39 versus 55	50 versus 57 versus 55	10.6 versus 11.3 versus NA	Simoa HD-X	NA	1.46 versus 1.43 versus 1.85	↓ in MSA-C ≈ SCAs < CTRL	^35^
**Friedreich’s ataxia**	Cross-sectional	Plasma	A versus CTRL	33 versus 13	61 versus 54	34 versus 37	20.4 versus NA	Simoa HD-1	1.33 versus 2.08	NA	No differences	^36^
	Longitudinal	Plasma	PA + A	187	53	31	NA	Simoa HD-1	NA	NA	↑ in PA + A	^29^
**MSA-C**	Cross-sectional	Plasma	MSA-C versus SCAs versus CTRL	58 versus 29 versus 100	43 versus 45 versus 56	59 versus 47 versus 60	NA	Simoa HD-X	NA	1.4 versus 1.4 versus 2.0	↓ in MSA-C < CTRL MSA-C ≈ SCAs	^37^
	Cross-sectional	CSF	A versus CTRL	5 versus 5	60 versus 60	55 versus 51	20.7 versus 0.4	ELISA	80.43 versus 43.7	NA	↑ in A	^32^

Data are presented as mean or median. Values were rounded to the nearest integer (age, sex) or to one decimal place (SARA score, tau). For longitudinal studies, the data presented correspond to baseline. Demographic and clinical data (age, sex and SARA) are reported as provided in each manuscript; due to missing data, in many studies, the number of individuals characterized for age and/or SARA does not always correspond to the number of individuals included in the tau analysis.

A, ataxic; CTRL, control; MSA-C, multiple system atrophy—cerebellar phenotype; NA, not available; PA, preataxic; p-tau, phosphorylated tau; SARA, Scale for the Assessment and Rating of Ataxia; t-tau, total tau; yr, year/s.

^a^SCA1 (*n* = 2), SCA2 (*n* = 7), SCA3 (*n* = 27), SCA6 (*n* = 1), SCA7 (*n* = 1), SCA8 (*n* = 4), SCA36 (*n* = 1), other SCAs (*n* = 17).

**Table 4:** Summary of studies evaluating disease proteins levels in hereditary cerebellar ataxias

Disease	Type of study	Groups	Sample	Protein	*N*	Sex (female, %)	Age (years)	SARA score	Assay/platform	Protein (pg/mL)	Main findings	Ref
SCA3	Cross-sectional	PA versus A versus CTRL	Plasma	PolyQ ataxin-3	4 versus 41 versus 34	75 versus 65 versus 59	32 versus 53 versus 55	0 versus 12 versus 0	MSD	840 versus 1280 versus 0	↑ in PA and A > CTRL	^38^
		PA versus A versus CTRL	Plasma	PolyQ ataxin-3	2 versus 40 versus 30	100 versus 67 versus 87	38 versus 53 versus 43	2 versus 17 versus NA	MSD	1210 versus 1080 versus 0	↑ in PA and A > CTRL	^38^
		PA versus A versus CTRL	Plasma	PolyQ ataxin-3	11 versus 45 versus 15	55 versus 51 versus 47	35 versus 44 versus 50	0.7 versus 15.3 versus 0.2	SMC	54 versus 83.3 versus 0	↑ in A > PA > CTRL Correlates with severity	^39^
		PA versus A versus CTRL	CSF	PolyQ ataxin-3	4 versus 45 versus 33	75 versus 65 versus 59	32 versus 51 versus 45	NA	MSD	70 versus 130 versus 0	↑ in A > PA > CTRL	^38^
		PA versus A versus CTRL	CSF	PolyQ ataxin-3	5 versus 12 versus 18	80 versus 67 versus 56	35 versus 50 versus 44	1 versus 10.2 versus 0	SMC	6 (patients) versus 0	↑ PA ≈ A > CTRL	^39^
		PA versus A versus CTRL	PBMCs	PolyQ ataxin-3	4 versus 24 versus 31	25 versus 42 versus 36	30 versus 53 versus 44	0.7 versus 16.0 versus NA	TR-FRET	NA versus NA versus NA	↑ PA ≈ A > CTRL	^40^
		PA + A	PBMCs	PolyQ ataxin-3	10	NA	NA	NA	LICOR Western Blot	NA	Ratio expanded/normal correlates with repeat length and age of onset	^41^
	Longitudinal	PA versus A versus CTRL	Plasma	PolyQ ataxin-3	5 versus 28 versus 4	80 versus 54 versus 50	36 versus 52 versus 45	1 versus 12.5 versus 0.5	SMC	NA versus NA versus NA	No changes after 1 yr	^39^
		PA versus A versus CTRL	Plasma	PolyQ ataxin-3	14 versus 73 versus 10	57 versus 40 versus 60	34 versus 52 versus 45	1.0 versus 10.5 versus 0	SMC	364 versus 403 versus 5	Stable during disease course	^27^
Friedreich’s ataxia	Cross-sectional	HE versus HO versus CTRL	Blood	Frataxin-M	25 versus 25 versus 25	NA	NA	NA	UHPLC-MRM/MS	7530 versus 3110 versus 10 730	↓ in HO < HE < CTRL Correlates with repeat length and age of onset	^42^
		HE versus HO versus CTRL	Blood	Frataxin-E	25 versus 25 versus 25	NA	NA	NA	UHPLC-MRM/MS	14 110 versus 3690 versus 20 610	↓ in HO < HE < CTRL Correlates with repeat length and age of onset	^42^
		HE versus HO versus CTRL	Blood	Total frataxin	25 versus 25 versus 25	NA	NA	NA	UHPLC-MRM/MS	21 640 versus 6820 versus 31 340	↓ in HO < HE < CTRL. Correlates with repeat length and age of onset	^42^
		A versus CTRL	Platelets	Total frataxin	7 versus 7	57 versus 57	NA	NA	UHPLC-PRM/MS	2 versus 9 (pg/µg)	↓ in A < CTRL	^43^

Disease	Type of Study	Groups	Sample	Protein	*N*	Sex (female, %)	Age (years)	SARA score	Assay/Platform	Protein (pg/mL)	Main findings	Ref
Friedreich’s ataxia	Longitudinal	HO versus CTRL	Blood	Frataxin-M	100 versus 11	60 versus 55	NA	NA	UHPLC-PRM/MS	7500 versus 2100	↓ in HO < CTRL	^44^
		HO versus CTRL	Blood	Frataxin-E	100 versus 11	60 versus 55	NA	NA	UHPLC-PRM/MS	4700 versus 26 800	↓ in HO < CTRL	^44^
		HO versus CTRL	Blood	Total frataxin	100 versus 11	60 versus 55	NA	NA	UHPLC-PRM/MS	680 versus 34 200	↓ in HO < CTRL	^44^

Data are presented as mean or median. Values were rounded to the nearest integer (age, sex and protein) or to one decimal place (SARA score). For longitudinal studies, the data presented correspond to baseline. Demographic and clinical data (age, sex and SARA) are reported as provided in each manuscript; due to missing data, in many studies, the number of individuals characterized for age and/or SARA does not always correspond to the number of individuals included in the tau analysis.

A, ataxic; CTRL, control; HE, heterozygous; HO, homozygous; MSD, Meso Scale Discovery; NA, not available; PA, preataxic; PBMCs, peripheral blood mononuclear cells; SARA, Scale for the Assessment and Rating of Ataxia; SMC, single molecule counting; UHPLC-MRM/MS, ultrahigh-performance liquid chromatography-multiple reaction monitoring/mass spectrometry; UHPLC-PRM/MS, ultrahigh-performance liquid chromatography-parallel reaction monitoring/mass spectrometry; yr, year/s.

## Results

### Neurofilaments in healthy population

Under physiological conditions, serum NfL levels at birth are ∼7 pg/mL and remain stable at this level for approximately the first 4 years of life, reflecting ongoing neuronal migration and differentiation in the developing brain.^45,46^ The concentration then decreases by ∼6.8% per year, until reaching a plateau of ∼4 pg/mL around the age of 10.^45,46^ From the age of 30 onwards, NfL levels increase physiologically, rising from ∼5 to ∼17.5 pg/mL at 60 years, with an average increase of 3–4% per year (0.366 pg/mL).^47^ Thereafter, NfL markedly increases throughout the sixth and subsequent decades of life.^47^ CSF NfL levels also increase with age in adults.^48^ This positive correlation between NfL levels and age may reflect age-related neuronal loss.^47^ Because NfL increases with age, values must be interpreted with reference to the individual’s age rather than in isolation. Another factor that significantly influences NfL levels is renal function, with serum creatine positively correlating with plasma and serum NfL levels, especially in older individuals.^49^ The influence of sex on NfL levels remains less clearly established. While no significant sex-related differences have been reported for serum NfL,^47^ higher CSF NfL concentrations have been observed in men compared to women.^48^

### Neurofilaments in spinocerebellar ataxias

#### Polyglutamine spinocerebellar ataxias

##### Ataxic carriers

In the context of spinocerebellar ataxias (SCAs), initial studies have indicated elevated NfL levels in carriers compared to healthy controls. A pioneering pilot study involving 20 ataxic carriers of SCA1, SCA2, SCA3 and SCA6 reported higher serum NfL concentrations relative to control groups.^23^ Subsequent larger cross-sectional studies confirmed a correlation between NfL levels and clinical progression in both CSF and blood, particularly in SCA2^18^ and SCA3.^16,33,50,51^ Longitudinal studies assessing plasma NfL further validated its utility as a biomarker, showing significant differences between healthy controls (∼5–10 pg/mL) and polyglutamine (polyQ) SCA carriers: SCA1 (∼18–35 pg/mL), SCA2 (∼16–20 pg/mL), SCA3 (∼20–35 pg/mL) and SCA7 (∼22–26 pg/mL).^24,27,52,53^ NfL concentrations increase throughout the disease course in polyQ SCAs, although the rate of increase slows down after symptom onset, despite progression of ataxia and cerebral atrophy.^24,27,52,53^ Longitudinal studies demonstrated that NfL levels remain stable over a 2-year follow-up across several polyQ SCA subtypes,^24^ with only a modest increase observed over the entire disease course, from presymptomatic to symptomatic phase, in SCA3.^27^ Thus, elevated NfL levels appears to reflect active neurodegeneration but do not consistently increase as clinical symptoms progress after disease onset. At baseline, higher plasma NfL levels predict greater clinical progression and cerebellar volume loss at 2-year follow-up across several SCA subtypes.^18,24,50^

Some puzzling features remain. First, SCA3 individuals exhibit higher NfL concentrations than those with other SCAs, while they often show less severe clinical progression as measured by the Scale for the Assessment and Rating of Ataxia (SARA).^24,54^ This discrepancy may reflect the more pronounced peripheral nervous system involvement in SCA3 compared with other polyQ SCAs. A second consideration is that NfL levels appear to stabilize over time in SCAs, a pattern also observed in amyotrophic lateral sclerosis, frontotemporal dementia, and atypical parkinsonian syndromes.^1^ This ‘plateau’ effect might mask age-related increases in NfL levels. Finally, NfL concentrations in polyQ SCAs lie between the higher levels observed in amyotrophic lateral sclerosis or MSA and the lower levels observed in conditions, such as Friedreich’s ataxia or Parkinson’s disease,^1,48^ possibly reflecting differences in disease progression rates or in the extent of nervous system involvement.

##### Preataxic carriers

Studies on preataxic SCA carriers have shown that NfL levels begin to rise well before the onset of clinical symptoms. Significant differences compared to healthy controls have been observed for up to 5 years before the estimated age at onset in SCA1,^17,53^ up to 20 years in SCA3^27,51,53,55^ and ∼10 years in SCA2 and SCA7.^26^ These time-points precede volumetric MRI changes in SCA1, SCA2, SCA3 and SCA7.^26,27,51,55^ This is illustrated by the observation that even the most sensitive brain regions (assessed by MRI), such as the pons and the superior cerebellar peduncle, exhibit the earliest evidence of volume loss only ∼5 years before symptom onset.^17,26,27^ Thus, fluid NfL levels currently represent the most promising biomarker for characterizing the preataxic phase of SCAs.

NfL levels can distinguish preataxic carriers of polyQ SCAs from both healthy controls and symptomatic individuals. Reported plasma concentrations in presymptomatic carriers are intermediate, with approximate values of ∼15.5–18 pg/mL in SCA1, ∼15.5 pg/mL in SCA2, ∼15–47 pg/mL in SCA3 and ∼12–15.5 pg/mL in SCA7.^16-18,26,27,34,50,51,53^ Moreover, preataxic carriers exhibiting non-cerebellar features present higher NfL levels compared to both controls and presymptomatic carriers. This was reported for SCA7 carriers exhibiting pyramidal signs or reduced visual acuity,^24,56^ indicating that NfL is also sensitive to non-cerebellar manifestations. In SCA3, carriers with only non-cerebellar signs exhibit NfL concentrations (∼20 pg/mL) that lie between those of presymptomatic (∼12 pg/mL) and ataxic carriers (∼35 pg/mL),^27,50,53^ further supporting the association between NfL levels and disease severity, even at the earliest disease stages.

##### Correlations with other parameters

Some studies reported a moderate correlation between serum or plasma NfL levels and CAG repeat length across all polyQ SCA subtypes, as well as with clinical scores, such as SARA and the inventory of non-ataxic signs (INAS).^16,24,34,35,50,51^ However, other studies have reported, at best, weak correlations.^17,25^ Significant correlations have also been observed with MRI volumetric markers, including brainstem atrophy, particularly pronounced in SCA3,^16,24^ and cerebellar atrophy in SCA2^18^ and SCA3.^16^ In SCA3, serum NfL levels have additionally been associated with grey matter volume in the left precentral gyrus and paracentral lobule, as well as with increased mean diffusivity in widespread white matter tracts,^50^ suggesting a link with both cortical and subcortical neurodegeneration. In SCA7, serum NfL levels correlate with retinal degeneration, as measured by the outer nuclear layer thickness assessed by optical coherence tomography.^56^

#### SCA27B

While most studies have focused on the common polyQ ataxias, a recently discovered autosomal dominant ataxia, SCA27B, is proven to be quite prevalent.^57^ This ataxia is caused by an abnormal GAA expansion in intron 1 of the *FGF14* gene.^58^

Levels of NfL in SCA27B (∼16 pg/mL) tend to increase with age and disease duration,^52^ but are not significantly different from controls or sporadic ataxias.^59^ Plasma NfL levels tend to vary depending on the phenotype, with atypical GAA-*FGF14* ataxia (i.e. early onset ataxia and MSA-C-like phenotypes) having higher levels of NfL (∼27 pg/mL) compared to SCA27B individuals with late-onset ataxia (∼22 pg/mL).^60^ Moreover, individuals with atypical GAA-*FGF14* ataxia had similar NfL levels to individuals with SCA3 (∼30 pg/mL) and MSA-C (∼38 pg/mL).^60^ However, these results from Ouyang *et al*.^60^ should be interpreted cautiously due to the small GAA-*FGF14* sample size (*n* = 2 with early-onset ataxia, *n* = 6 with late-onset ataxia and *n* = 3 with MSA-C like phenotypes) and the GAA repeat size distribution bias. Indeed, the early-onset ataxia group had a longer GAA repeat size above 300 repeats, whereas only one individual with late-onset ataxia or MSA-C phenotypes had a longer allele above 300 GAA. Considering the incomplete penetrance associated with GAA expansion between 250 and 300 repeats,^57^ further studies focusing on larger alleles are warranted.

### Neurofilaments in autosomal recessive ataxias

#### Friedreich’s ataxia

Friedreich’s ataxia is the most common autosomal recessive ataxia caused by a GAA expansion in the first intron of the *FXN* gene.^61^ Serum levels of NfL are elevated in individuals with Friedreich’s ataxia compared to healthy controls, both in childhood and adulthood.^28,36,62,63^ In contrast, heterozygous carriers exhibit levels similar to controls.^30^ Unlike the age-related increase observed in healthy individuals, NfL levels in Friedreich’s ataxia decrease over time, with a reported reduction of 3.5 pg/mL per year in paediatric patients.^28^ This decline becomes particularly evident after the age of 40, when NfL levels approach those of age-matched controls (∼22 pg/mL).^29,30^ The trajectory of NfL levels in Friedreich’s ataxia is distinct from adult-onset polyQ SCAs,^24^ with concentrations peaking around disease onset and gradually decreasing in advanced stages, a pattern also observed in other paediatric neurodegenerative diseases, such as spinal muscular atrophy (SMA).^46^

The association between blood NfL levels and clinical features of Friedreich’s ataxia is inconsistent across studies. While some reports indicate correlations with age at onset and disease severity,^30^ others have not observed such associations.^28,29,31^ NfL levels are inversely correlated with the GAA repeat length on the longer allele, suggesting lower NfL concentrations in the most severe, early-onset forms of Friedreich’s ataxia.^29,31^ This inverse correlation might be attributed to advanced neurodegeneration, cellular adaptation processes, growth failure or hypoplasia/atrophy of neurological tissue in severe cases, potentially leading to a progressively reduced neuronal mass capable of releasing NfL.^29^

#### CANVAS

Cerebellar ataxia, neuropathy and vestibular areflexia syndrome (CANVAS) is caused by an expanded pentameric repeat (AAGGG) in intron 2 of the *RFC1* gene.^64^ Although recently discovered, it is increasingly recognized as one of the more common recessive causes of late onset ataxia. Only one study has assessed serum NfL levels in CANVAS, reporting higher concentrations in patients with isolated sensory neuropathy (∼24 pg/mL) compared to controls (∼12 pg/mL), and higher levels in more advanced disease stages with concurrent cerebellar involvement associated with neuropathy (∼28 pg/mL) compared to patients with isolated sensory neuropathy.^65^

### Spastic paraplegia type 7

Spastic paraplegia type 7 (SPG7), also known as hereditary spastic paraplegia type 7 (HSP7), is a typical autosomal recessive disease caused by mutations in the *SPG7* gene. It is characterized by a variable involvement of the corticospinal tracts and cerebellar pathways, resulting in a clinical spectrum that ranges from pure spastic paraplegia to pure cerebellar ataxia, with many patients exhibiting a mixed spastic-ataxia phenotype.^66^ One study reported NfL levels of 10.4 pg/mL in patients with SPG7 with a mean age at sampling of ∼58 years.^52^ These values are similar to those reported for age-matched controls.^47^ In another study including different HSPs, among which 11 patients with SPG7 were included out of a total of 63 patients, serum NfL levels were increased in HSP individuals compared to controls (24.7 pg/mL versus 18.9 pg/mL) and increased with disease progression. However, HSP genotype-specific levels were not reported.^23^

### Neurofilaments in multiple system atrophy

Although MSA is not a hereditary cerebellar ataxia, we included it because it is frequently used for comparison in SCA studies. Serum NfL levels are significantly elevated in individuals with MSA cerebellar type (MSA-C) compared to controls, with reported values typically ranging from ∼29 to 40 pg/mL.^23,35,37,60,67-69^ No significant differences in NfL levels were observed between the cerebellar (MSA-C) and parkinsonian (MSA-P) subtypes.^37,69^ The utility of NfL for discriminating MSA-C from sporadic late-onset ataxia appears variable across studies and is likely influenced by the heterogeneous underlying aetiologies of sporadic late-onset ataxia.^23,67,68^ Furthermore, NfL levels did not differ between MSA-C and various SCAs, including SCA1, SCA2, SCA3, SCA6, SCA7, SCA8, SCA36 and rarer forms,^35,37^ except for SCA27B, in which NfL levels are significantly lower than in MSA-C.^59^

Cross-sectional analyses consistently showed that higher baseline NfL levels are associated with greater disease severity, as measured by clinical scales, higher mortality rate^37,69-71^ and reduced striatal volume.^69^ However, baseline NfL levels could not distinguish individuals who already met diagnostic criteria for MSA at baseline from those who later fulfilled criteria during follow-up, indicating clinical conversion.^68^ Longitudinal analysis revealed a mild decrease in NfL in MSA-C, whereas its levels did not change in sporadic adult-onset ataxia.^68^ More specifically, another longitudinal MSA study showed a biphasic trajectory of NfL levels, characterized by an initial slow increase, followed by an accelerated rise, which then plateaued into a second slower phase after ∼8 years of disease progression.^69^

Collectively, these findings underscore the value of NfL as a biomarker of disease severity and predictor of shorter survival in MSA-C. This supports its potential utility as an objective outcome measure for assessing neuronal protection and tracking disease progression in clinical trials.^69^

### Neurofilament potential in clinical trials

#### Monitoring safety and efficacy

According to the literature reviewed above, NfL appears to be the most promising candidate for near-term implementation in clinical trials, especially for polyQ SCAs. NfL levels are validated biomarkers of neuroaxonal injury, and the advent of highly sensitive quantification methods has enabled reliable measurement in blood samples simplifying longitudinal monitoring. This advancement facilitates the integration of NfL into clinical practice. Potential applications include the assessment of disease activity, especially in preataxic stage, monitoring of treatment responses and use as a stratification biomarker for patients’ selection. The utility of NfL is well illustrated by its application in other neurological disorders. For instance, the approval of tofersen, an antisense oligonucleotide (ASO) for *SOD1*-associated amyotrophic lateral sclerosis, was supported by a pronounced reduction in serum NfL levels, with median values decreasing from 78 to 36 pg/mL during treatment.^72,73^ Similarly, in SMA, treatment with nusinersen leads to a decrease in NfL levels, which are elevated in both serum (∼585 pg/mL) and CSF (∼11 pg/mL) of adult patients compared to controls.^74,75^ This reduction has been correlated with both patient age and clinical improvements.^76^ These precedents provide rationale for incorporating NfL as a key pharmacodynamic endpoint in future SCA trials.

The dual role of NfL in monitoring both efficacy and safety of therapeutic interventions is exemplified by experience in Huntington’s disease. In a Phase 1–2a trial of tominersen, an ASO targeting mutant *HTT* mRNA, an increase in CSF NfL was observed in the 90 and 120 mg treated cohorts after 1 or 2 months upon intrathecal injection, respectively, with values returning to baseline levels (i.e. prior to the intervention) after 7–27 months.^77^ Nevertheless, the subsequent Phase 3 trial (NCT03761849) was halted due to clinical worsening in treated participants, including ventricular enlargement, highlighting how NfL elevations can serve as an early indicator of adverse biological activity.

### Stratification of participants

Stratifying participants based on genetic characteristics (i.e. CAG repeats length) and biomarkers of disease activity, such as NfL, is critical for demonstrating therapeutic efficacy in SCA trials. For instance, in SCA1, higher NfL levels in preataxic individuals predict a shorter time to symptom onset, allowing for the identification of those most likely to progress during a trial period.^17^ Therefore, NfL levels could be used as an inclusion criterion to enrich trials with a population at higher risk of progression. This approach is already being implemented in other neurodegenerative diseases.^78,79^ The Phase 3 study of latozinemab in frontotemporal dementia due to *GRN* pathogenic variants used elevated serum NfL to define an at-risk cohort.^78^ Similarly, the ATLAS study of tofersen in presymptomatic *SOD1* carriers utilized elevated serum NfL as a biomarker of underlying disease activity to initiate treatment.^79^

### Surrogate endpoint in preataxic phase

NfL has significant potential as a surrogate endpoint in trials for preataxic carriers, in whom clinical symptoms are absent. Given that NfL levels rise years before symptom onset, a treatment-induced reduction in NfL could serve as an objective biological measure of a drug’s effect on the underlying neurodegenerative process. This could enable the evaluation of efficacy even at the individual participant level. Furthermore, serum or plasma NfL levels, potentially in combination with CAG repeat length, could support individualized management and counselling of preataxic carriers by providing insight into their biological disease stage and proximity to clinical onset. This may help to identify individuals with the greatest urgency for initiating disease-modifying therapies.

### Other biomarkers in ataxias

#### Glial fibrillary acidic protein

GFAP is a Type III intermediate filament protein predominantly expressed in astrocytes. Following astrocytic damage or activation, GFAP is released into the CSF and blood, where its quantification has been made reliable by ultra-sensitive assays, such as Simoa.^80^ Elevated GFAP levels reflect CNS injury and/or inflammation, as observed in traumatic brain injury, multiple sclerosis or stroke.^81^ GFAP concentrations also increase with age.^82^ The direct link between GFAP dysregulation and neurodegeneration is exemplified by Alexander disease, a leukodystrophy caused by pathogenic variants in the *GFAP* gene. In this disorder, mutant *GFAP* leads to toxic protein accumulation, astrocyte dysfunction and subsequent CNS degeneration.^83^ Although *GFAP* is not a direct causative gene in ataxias, its biological relevance and potential as a biomarker are supported by several key observations.

A pilot study revealed elevated CSF GFAP levels in patients with MSA-C (∼710 pg/mL) and SCA2 (∼680 pg/mL) compared to controls (∼290 pg/mL), but not in SCA1 (∼360 pg/mL) and SCA6 (∼310 pg/mL).^32^ Subsequent research has highlighted this heterogeneity. For instance, in SCA3, one study reported increased serum GFAP in ataxic patients (∼8900 pg/mL) compared to controls (∼3800 pg/mL),^22^ while another study did not find any differences between preataxic, ataxic and control groups.^33^ Similarly, a longitudinal study in SCA1 reported higher CSF GFAP levels in patients (∼1800pg/mL) than in controls (∼690 pg/mL), whereas plasma levels were comparable (125.8 versus 106.8 pg/mL) and correlated with age but not with disease severity or CAG expansion length.^25^ In SCA7, elevated GFAP levels in both plasma and CSF were observed in affected individuals (plasma, 82.7 versus 48 pg/mL; CSF, 9300 versus 8900 pg/mL), with even higher values in three preataxic carriers (plasma, 62.4 pg/mL; CSF, 14 900 pg/mL).^34^ Conversely, a large cross-sectional study reported that plasma GFAP levels were significantly higher in MSA-C patients compared to a mixed cohort of SCAs (i.e. SCA1, SCA2, SCA3, SCA6, SCA7, SCA8, SCA36 and rarer SCAs; ∼95 versus 69 pg/mL), but GFAP levels in SCAs did not significantly differ from controls (∼59 pg/mL).^35^ This pronounced GFAP elevation in MSA-C compared to controls (∼98 versus 69 pg/mL) has been corroborated by another study.^37^ In Friedreich’s ataxia, GFAP concentrations are reported elevated compared to controls (∼99 versus 60 pg/mL), correlating with NfL levels, and are particularly increased in individuals under 15 years of age.^29,36^

Astrocytes play a crucial role in cerebellar function and neuroinflammation, which can be part of the pathological cascade in many hereditary ataxias (e.g. SCAs and Friedreich’s ataxia). Elevated GFAP levels in CSF or blood in hereditary ataxias may reflect reactive astrogliosis, a hallmark of neurodegeneration, which appears later in the disease.^84^ By contrast, NfL elevation reflects the degeneration of demyelinated axons, which is an important early pathological change in hereditary ataxias. This temporal distinction suggests that GFAP and NfL could serve as complementary biomarkers, with NfL potentially more sensitive for initial stages and GFAP reflecting subsequent glial responses. However, most of the current evidence is cross-sectional, so the temporal dynamics of GFAP remain largely theoretical. Further longitudinal studies in larger, well-characterized cohorts are needed to fully evaluate its utility as a prognostic or monitoring biomarker for hereditary ataxias.

#### Tau and phosphorylated tau

The microtubule-associated protein tau, encoded by the *MAPT* gene, is a well-established biomarker for neurodegenerative diseases, such as Alzheimer’s disease,^85^ Creutzfeldt–Jakob disease^86^ and Huntington’s disease.^87^ However, its utility in hereditary ataxias appears more limited and complex. Initial studies reported variable changes in tau levels across different ataxias. CSF t-tau levels were elevated in SCA2 (∼110 pg/mL) and MSA-C (∼80 pg/mL) compared to controls (∼44 pg/mL).^32^ In SCA3, intriguingly, CSF t-tau and p-tau levels were found to be higher in preataxic carriers (t-tau, ∼384 pg/mL; p-tau, ∼51 pg/mL) than ataxic patients (t-tau, ∼227 pg/mL; p-tau, ∼32.5 pg/mL), and plasma t-tau was elevated in young carriers (<40 years) but decreased with age more steeply than in controls.^33^ In contrast, other studies found no differences in plasma and CSF t-tau and p-tau181 levels in preataxic and early ataxic SCA2 (plasma t-tau, ∼5.9 pg/mL; plasma p-tau181, ∼1.7 pg/mL; CSF t-tau, ∼128 pg/mL; CSF p-tau181, ∼26.7 pg/mL) and SCA7 carriers (plasma t-tau, ∼6.8 pg/mL; plasma p-tau181, ∼1.4 pg/mL; CSF t-tau, ∼136 pg/mL; CSF p-tau181, ∼30.8 pg/mL), which were similar to controls (plasma t-tau, ∼6.5 pg/mL; plasma p-tau181, ∼1.5 pg/mL; CSF t-tau, ∼116 pg/mL; CSF p-tau181, ∼27 pg/mL).^26^ No longitudinal changes over 1 year were observed. Studies exploring tau levels in SCAs and MSA reported lower p-tau plasma levels in both MSA-C (∼1.4 pg/mL) and SCA (∼1.4–1.5 pg/mL) individuals, including SCA1, SCA2, SCA3, SCA6, SCA7, SCA8, SCA36 and rarer SCAs, compared to controls (∼1.85–2 pg/mL).^35,37^ In Friedreich’s ataxia, findings on plasma t-tau levels are conflicting. One study reported no significant differences (∼1.3 versus ∼2.1 pg/mL),^36^ whereas another described a moderate increase.^29^ In both studies, the same platform (Simoa HD-1) was used for analysis.

Collectively, these findings suggest that tau assays have limited prognostic utility in hereditary ataxias. This can be attributed to several factors. First, tau is a marker of damage to thin, unmyelinated axons, which are not specifically affected in many ataxias.^36^ Second, the interpretation of plasma tau levels is complicated by its peripheral origins; only an estimated 20% of plasma t-tau is brain derived, with the rest originating from tissues, such as the liver and kidney.^88,89^ Consequently, plasma t-tau may be more relevant in acute CNS injuries than in chronic, slowly progressive diseases, such as inherited ataxias.^90^ Furthermore, the presence of different tau isoforms, including six CNS-specific variants distinguishable from peripheral isoforms by alternative splicing, adds another layer of complexity.^91^ To overcome these limitations, a novel biomarker known as brain-derived tau (BD-tau) has been developed. BD-tau assays are designed to be specific to CNS isoforms. In Alzheimer’s disease, BD-tau effectively discriminates patients from controls, and plasma BD-tau levels correlate strongly with CSF BD-tau and NfL levels.^90^ The potential of this promising biomarker has not yet been explored in hereditary ataxias.

#### Disease proteins

PolyQ SCAs, including SCA1, SCA2, SCA3, SCA6 and SCA7, are caused by repeat expansions in their respective genes (*ATXN1*, *ATXN2*, *ATXN3*, *CACNA1A*, *ATXN7*), leading to the production of toxic mutant proteins. Pharmacological strategies aimed at lowering the expression of these mutant proteins in the brain represent a promising therapeutic approach that targets the underlying disease mechanism. Consequently, the direct quantification of mutant ataxin proteins in accessible biofluids is a critical objective for demonstrating target engagement in future clinical trials.

In SCA3, the quantification of polyQ-expanded ataxin-3 has been pioneered using various immunoassays. An initial study employing an electrochemiluminescence immunoassay (MSD system) detected polyQ-expanded ataxin-3 in the CSF, plasma and fibroblast of SCA3 individuals, distinguishing them from controls in which levels were undetectable (CSF, 0.00013 pg/mL; plasma, 0.00128 pg/mL; fibroblast, 0.00456 pg/mL).^38^ The lack of correlation between CSF and plasma levels, along with a 10-fold higher concentration in plasma, suggests distinct pools of polyQ-expanded ataxin-3 protein in these compartments.^38^ PolyQ expanded ataxin-3 was later on also detected in urine of SCA3 individuals using MSD platform.^80^ Subsequent studies using a single molecule counting immunoassay confirmed significantly higher plasma polyQ-expanded ataxin-3 levels in ataxic individuals compared to preataxic carriers (∼83 versus 54 pg/mL^39^; ∼403 versus 360 pg/mL^27^), while levels remained undetectable in controls.^27,39^ In longitudinal studies, it was observed that concentrations of polyQ ataxin-3 remained stable throughout the entire disease course, and no significant correlations were found with disease severity, CAG repeat length or age at onset.^27,38^ The nearly constant levels observed in SCA3 carriers from the preataxic stage position polyQ-expanded ataxin-3 as a robust biomarker for target engagement rather than for tracking disease progression.

Furthermore, a time-resolved fluorescence energy transfer (TR-FRET) immunoassay can detect polyQ-expanded ataxin-3 protein in peripheral blood mononuclear cells, with severe patients exhibiting higher levels of polyQ ataxin-3 compared to moderate, mild or preataxic individuals^40^ and alterations in the ratio of expanded to normal ataxin-3 levels correlating with repeat length and age of onset.^41^ However, this technique has proven insufficiently sensitive for detecting polyQ proteins in biofluids.^40^

Beyond SCA3, a polyQ-expanded ataxin-2-specific TR-FRET immunoassay has been established and validated in SCA2 patient’s fibroblasts and neurons derived from induced pluripotent stem cells, but showed no sensitivity to detect soluble protein in serum from SCA2 patients.^92^ Ultra-sensitive assays for measuring SCA2 have not yet being reported. Friedreich’s ataxia is caused by a deficiency in the frataxin protein. Consequently, a primary goal of many pharmacological therapies is to increase frataxin levels.^93^ Since frataxin is not secreted into circulation, it cannot be reliably measured in serum or plasma. However, whole blood serves as a valuable source, as distinct frataxin isoforms are expressed in different cell populations: mitochondrial frataxin (frataxin-M) in nucleated cells and extramitochondrial frataxin (frataxin-E) in erythrocytes.^43^ Studies using stable isotope dilution ultrahigh-performance liquid chromatography have quantified these isoforms, consistently reporting decreased levels of frataxin-M, frataxin-E and total frataxin in individuals with Friedreich’s ataxia compared to controls.^42-44^ All frataxin levels were inversely correlated with GAA repeat length and age at onset, with frataxin-E generally showing stronger correlations than frataxin-M.^42^ Collectively, these studies validate the measurement of frataxin isoforms in whole blood, establishing them as key biomarkers for tracking the pharmacological response to interventions aimed at increasing frataxin expression.

In conclusion, these data support the use of disease-associated proteins as valuable target-engagement biomarkers, reinforcing their utility in the assessment of gene-targeted therapeutic strategies.

#### Somatic repeat expansion

Somatic instability is a well-recognized feature of repeat expansion disorders and refers to the progressive, tissue-specific changes in repeat length that occur post-zygotically, leading to somatic mosaicism within an individual. Accumulating evidence suggests that DNA repair pathways play a central role in driving repeat expansion dynamics, thereby contributing to inter-individual variability and modifying disease courses.^94^ Recently, Handsaker *et al*.^95^ demonstrated that in Huntington’s disease (a polyQ disease), cell death exhibits marked cell-type specificity, preferentially affecting striatal projection neurons once the CAG repeat length surpasses a toxicity threshold, leading to rapid progression towards neuronal degeneration. Therefore, the extent of somatic repeat expansion may drive disease progression and could serve as a dynamic biomarker of pathogenic advancement.

In the polyQ SCAs driven by exonic CAG repeat expansion (i.e. SCA1, SCA2, SCA3, SCA6, SCA7, SCA17 and DRPLA), somatic instability is both gene and tissue specific and increases throughout life. Indeed, longitudinal analyses of blood samples collected for a mean of 8 years showed that CAG repeat size increased over time, with the greatest increases observed in SCA7 and the slowest in SCA3. These increases correlate with repeat length and disease severity. Brain tissues in adults exhibit larger somatic expansions than peripheral blood. Interestingly, the cerebellum shows the lowest degree of mosaicism compared to other central nervous system regions, such as the striatum or brainstem. Also, foetal tissues carrying CAG expansion responsible for SCA1, SCA3 and SCA7 have been explored, showing no detectable somatic expansion in foetal cortices or other CNS regions at mid-gestation (Gestational Week 13), indicating that somatic instability arises progressively during life.^96^

By contrast, SCA27B shows a different pattern: peripheral tissues, including serial blood samples collected over >20 years, exhibit minimal somatic instability, whereas post-mortem analysis reveals a marked cerebellar specific expansion bias, with levels of somatic expansion in the cerebellar hemispheres and vermis ∼2.7–3.2fold greater than in other brain regions.^97^

## Conclusion

Fluid biomarkers are essential clinical trial design, particularly in studies targeting the preataxic phase. NfL is the most extensively investigated biomarker with consistent reproducible results in several studies conducted in polyQ SCAs (e.g. SCA1, SCA2, SCA3 and SCA7), SCA27B, MSA-C and recessive ataxias (Friedreich’s ataxia, CANVAS, SPG7). Its elevation in blood years before clinical onset confirms that axonal neurodegeneration is an early and primary event in disease pathogenesis. Correlations with brain MRI volumetric measures and disease severity, as assessed by clinical scales, such SARA and INAS, further support its potential utility as a progression biomarker for these diseases.

GFAP, although less extensively studied, may provide complementary information by reflecting astroglial activation and neuroinflammatory processes, which tend to occur later in the disease course. In contrast, tau has shown more heterogeneous results, likely reflecting differences across ataxia subtypes, analytical platforms and variable involvement of cortical and subcortical neuronal populations. Moreover, the progressive somatic CAG repeat instability observed in polyQ ataxias introduces an additional layer of molecular progression, which could help explain individual variability in disease course and may serve as a mechanistic biomarker if reliably quantified. For the assessment of target engagement, the quantification of disease-associated proteins using ultra-sensitive assays has emerged as a valuable approach, especially for the evaluation of gene-targeting therapies.

Despite these advances, several challenges remain that currently limit the clinical implementation of these biomarkers. First, the marked genetic and phenotypic heterogeneity of hereditary ataxias complicates the establishment of universal reference ranges. Second, the limited number of large-scale longitudinal studies and the lack of methodological harmonization across biomaterials analysed, biosampling procedures, and analytical platforms hinder direct comparisons and the definition of clinically meaningful cut-off values. Furthermore, most available data derive from observational studies, emphasizing the need to validate these biomarkers as outcome measures in interventional clinical trials. Finally, the temporal dynamics of different biomarkers (e.g. early NfL rise versus later GFAP increase) require integrated longitudinal approaches to fully understand their predictive and monitoring value.

In this context, the ‘Molecular Biomarkers & Biosampling’ Working Group of the Ataxia Global Initiative (AGI) has been actively working to provide guidance to the scientific community engaged in ataxia research, with the aim of harmonizing protocols and fostering the standardization of procedures across studies.^98^

Looking ahead, the integration of NfL into multimodal biomarker frameworks, in combination with clinical, neuroimaging and other molecular data, will be critical for a more comprehensive characterization of the natural history of hereditary ataxias. Such integrative approaches could enhance patient stratification, allow the identification of optimal therapeutic windows and inform the development and evaluation of disease-modifying therapies. In this context, fluid biomarkers represent a pivotal step towards more precise, personalized and translational research in hereditary ataxias, ultimately bringing the gap between mechanistic understanding and clinical application.

## Supplementary Material

fcag313_Supplementary_Data

## Data Availability

Data sharing is not applicable as no new data were created or analysed.
