# Supplementary material for: The role of neurofilament light chain and other fluid biomarkers in cerebellar ataxias
Source: Brain Commun. 2026 Oct 6;8(5):fcag313. doi: 10.1093/braincomms/fcag313 (PMC13639701; doi:10.1093/braincomms/fcag313)
Supplement: Supplementary file 1 [file fcag313_supplementary_data.zip › AGI_WG2_Biomarkers_Study_Group.pdf]

AGI-WG2-Biomarkers Study Group:

Anna Sulek[1], Astrid Gómez[2], Brent L. Fogel[3], Carmen Espinós[4], Cheick Cisse[5], Chiara Pane[6], Francesco Saccà[6], Gabriella Silvestri[7], Georg Auburger[8], Guido Primiano[9], Hayley S. McLoughlin[10], Jeannette Hübener-Schmid[11], Karen Anthony[12], Kevin D'Ostilio[13], Liana Veneziano[14], Lucie Stovickova[15], Luis E. Almaguer-Mederos[16], Maria Luiza Saraiva-Pereira[17], Mohammed Faruq[18], Nicolas Dupré[19], Norlinah Mohamed Ibrahim[20], Paula Pérez-Torre[21], Penina Ponger[22], Rui Jorge Nobre[23], Sheng-Han Kuo[24], Sylvia Boesch[25], Thorsten Schmidt[26], Vito Luigi Colona[27]

1. Faculty of Medicine, Lazarski University, Warsaw, Poland
2. Unidad de trastornos del movimiento. Servicio de Neurología. Hospital Virgen del Rocío. Sevilla.
3. Departments of Neurology and Human Genetics, David Geffen School of Medicine at the University of California, Los Angeles (UCLA). Los Angeles, CA, USA.
4. Genetics Department, Universitat de València - INCLIVA & CIBERER, Valencia, Spain
5. University of Sciences Techniques and Technologies of Bamako, Mali
6. GENESIS Department, Federico II University, Naples, Italy
7. Neurology Unit, Dipartimento di Neuroscienze, Organi Di Senso EeTorace, Fondazione Policlinico Universitario A Gemelli IRCCS, Rome, Italy
8. Institute for Clinical Neuroanatomy, Dr. Senckenberg Anatomy, Fachbereich Medizin, Goethe University Frankfurt, 60528 Frankfurt am Main, Germany
9. Fondazione Policlinico Universitario Agostino Gemelli IRCCS, Rome, Italy
10. Department of Neurology, University of Michigan, Ann Arbor, MI, USA
11. Department of Life Sciences, Reutlingen University, Reutlingen, Germany & Institute of Medical Genetics and Applied Genomics, University of Tuebingen, Tuebingen Germany
12. Centre for Physical Activity and Life Sciences, University of Northampton, Northampton, NN1 5PH, United Kingdom
13. UCB, Braine-L'Alleud, Belgium
14. IRCCS San Raffaele, Rome Italy
15. Department of Paediatric Neurology, Second Faculty of Medicine, Charles University, Motol University Hospital, Prague, Czech Republic
16. Department of Neuromuscular Diseases, Queen Square Institute of Neurology, London, United Kingdom
17. Biochemistry Department, Universidade Federal do Rio Grande do Sul, Porto Alegre, Brazil
18. CSIR-Institute of Genomics and Integrative Biology, Delhi, India
19. Neuroscience research program, CHU de Quebec-Université Laval, Quebec, Canada & Facutly of medicine, Dept. of medicine, Quebec, Canada
20. Neurology Unit, Department of Medicine, Faculty of Medicine, National University of Malaysia, Kuala Lumpur, Malaysia
21. CSUR for ataxias and hereditary spastic paraplegia. Department of Neurology. Ramón y Cajal Hospital. Madrid, Spain.
22. Genetics Service, Austin Hospital, Melbourne, Austrlia & Ataxia Clinic, Royal Victoria Eye and Ear Hospital, Melbourne Australia
23. Center for Neuroscience and Cell Biology, University of Coimbra, Portugal
24. Columbia University Medical Center
25. Center for rare movement disorders Innsbruck, Medical University Innsbruck, AUSTRIA
26. Institute of Medical Genetics and Applied Genomics, University of Tuebingen, Tuebingen, Germany
27. Research Unit of Neurorehabilitation, Bambino Gesù Children's Hospital, IRCCS, Rome, Italy
