# Supplementary material for: The role of neurofilament light chain and other fluid biomarkers in cerebellar ataxias
Source: Brain Commun. 2026 Oct 6;8(5):fcag313. doi: 10.1093/braincomms/fcag313 (PMC13639701; doi:10.1093/braincomms/fcag313)
Supplement: Supplementary file 1 [file fcag313_supplementary_data.zip › Supplementary_Materials.pdf]

Supplementary Table 1. Summary of studies evaluating NfL levels in hereditary cerebellar ataxias and MSA-C.

Disease	Type of study	Sample	Groups	N	Sex (female, %)	Age (years)	SARA score	Assay/ Platform	NfL (pg/mL)	Main findings	Ref
**SCA1**	Cross-sectional	Plasma	PA vs A vs CTRL	11 vs 26 vs 34	71 vs 55 vs 46	39 vs 47 vs 38	1.0 vs 9.0 vs 0	Simoa HD-1	18 vs 29 vs 6	↑ in A > PA > CTRL<br>↑ before onset	34
		Plasma	PA+A vs CTRL	54 vs 38	NA	45 vs 39	5.8 vs 0.7	Simoa HD-1	18 vs 5	↑ in PA+A	33
		CSF	PA vs A vs CTRL	1 vs 5 vs 89	100 vs 60 vs 48	NA	NA	ELISA	1030 vs 2971 vs NA	↑ PA and A > CTRL	17
	Longitudinal	Plasma	PA+A vs CTRL	17 vs 19	NA	45 vs 50	10.7 vs 1.1	Simoa HD-1	25 vs 10	↑ in PA+A<br>Cut-off to A = 16.87 pg/mL<br>No changes after 2 yr	32
		Plasma	PA+A vs CTRL	34 vs 20	45 vs 43	51 vs 50	13.0 vs 0	ELLA	35 vs 13	↑ in PA+A.<br>No changes after 1 yr	42
		Serum	PA vs A vs CTRL	23 vs 17 vs 89	74 vs 47 vs 55	26 vs 45 vs 31	0.5 vs 12.5 vs NA	Simoa HD-X	16 vs 32 vs 6	↑ in A > PA > CTRL<br>↑ in PA closer to onset<br>No changes after 2.7 yr	17
		CSF	PA+A vs CTRL	21 vs 21	45 vs 43	NA	12.5 vs 0	ELLA	3169 vs 739	↑ in PA+A<br>No changes after 1 yr	42
**SCA2**	Cross-sectional	Plasma	PA+A vs CTRL	26 vs 38	NA	43 vs 39	8.8 vs 0.7	Simoa HD-1	16 vs 5	↑ in PA+A	33
		Serum	A vs CTRL	23 vs 20	39 vs 50	40 vs 46	11.1 vs NA	Simoa HD-X	28 vs 7	↑ in A	18
		Serum	PA vs A vs CTRL	10 vs 49 vs 92	80 vs 39 vs 53	28 vs 41 vs 41	0 vs 10.6 vs NA	Simoa HD-X	16 vs 26 vs 7	↑ in A > PA > CTRL<br>Associated with severity	18
		CSF	A vs CTRL	23 vs 20	39 vs 50	40 vs 46	11.1 vs NA	Simoa HD-X	2188 vs 361	↑ in A	18
	Longitudinal	Plasma	PA+A vs CTRL	13 vs 19	NA	45 vs 50	13.3 vs 1.05	Simoa HD-1	20 vs 10	↑ in PA+A.<br>Cut-off to A = 19.1 pg/mL<br>No changes after 2 yr	32
		Plasma	PA+A vs CTRL	5+10 vs 10	33 vs 40	41 vs 40	4 vs 0	Simoa HD-1	14 vs 5	↑ in PA+A<br>No changes after 1 yr	38
**SCA3**	Cross-sectional	Plasma	PA vs A vs CTRL	20 vs 198 vs 185	60 vs 48 vs 51	34 vs 43 vs 42	1 vs 13 vs NA	Simoa HD-1	22 vs 36 vs 8	↑ in A > PA > CTRL	29
		Plasma	PA vs A vs CTRL	4 vs 41 vs 34	75 vs 65 vs 59	32 vs 53 vs 55	0 vs 12 vs 0	Simoa HD-1	15 vs 30 vs 11	↑ in A > PA > CTRL	85
		Plasma	PA vs A vs CTRL	2 vs 40 vs 30	100 vs 67 vs 87	38 vs 53 vs 43	2 vs 17 vs NA	Simoa HD-1	24 vs 32 vs 8	↑ in A > CTRL	85
		Plasma	PA vs A vs CTRL	23 vs 120 vs 172	57 vs 58 vs 53	35 vs 52 vs 49	1.0 vs 13.5 vs NA	Simoa HD-1	15 vs 26 vs 10	↑ in A > PA > CTRL	31
		Plasma	PA vs A vs CTRL	24 and 64 vs 39	64 vs 51 vs 46	36 vs 49 vs 38	1.0 vs 7.0 vs 0	Simoa HD-1	20 vs 31 vs 6	↑ in A > PA > CTRL	34

Disease	Type of study	Sample	Groups	N	Sex (female, %)	Age (years)	SARA score	Assay/ Platform	NfL (pg/mL)	Main findings	Ref
**SCA3**	Cross-sectional	Plasma	PA+A vs CTRL	122 vs 38	NA	46 vs 39	6.4 vs 0.7	Simoa HD-1	20 vs 5	↑ in PA+A	33
		Serum	A vs CTRL	17 vs 9	56 vs 65	50 vs 54	13.8 vs NA	Simoa HD-1	42 vs 9	↑ in A	16
		Serum	PA vs A vs PA or A CTRL	26 vs 90 vs 21 or 70	65 vs 54 vs 62 or 56	31 vs 44 vs 32 or 43	0.7 vs 11.7 vs NA or NA	Simoa HD-1	15 vs 38 vs 7 or 9	↑ in A > PA > CTRL<br>Cut-off to A = 20 pg/mL	16
		Serum	PA vs A vs CTRL	8 vs 75 vs 77	NA	34 vs 52 vs 44	1.0 vs 13.0 vs NA	Simoa HD-1	29 vs 35 vs 9	↑ in A ≈ PA > CTRL<br>Cut-off to A = 20.3 pg/mL<br>↑ 7.5 yr before onset	30
		Serum	PA vs A vs CTRL	14 vs 27 vs 48	NA	37 vs 51 vs 41	1.0 vs 11.5 vs NA	Simoa HD-1	47 vs 86 vs 19	↑ in A > PA > CTRL<br>Cut-off to A = 20.3 pg/mL<br>↑ 7.5 yr before onset	30
		CSF	A vs CTRL	17 vs 9	56 vs 65	50 vs 54	13.8 vs NA	Simoa HD-1	4262 vs 472	↑ in A	16
	Longitudinal	Plasma	PA+A vs CTRL	19 vs 19	NA	51 vs 50	14.1 vs 1.05	Simoa HD-1	35 vs 10	↑ in PA+A<br>Cut-off to A= 16.04 pg/mL<br>No changes after 2 yr	32
		Plasma	PA vs A vs CTRL	32 vs 179 vs 92	56 vs 50 vs 60	34 vs 52 vs 45	1.0 and 13.0 vs 0	Simoa HD-X	17 vs 27 vs 7	↑ 20 years before onset	35
**SCA7**	Cross-sectional	Plasma	PA+A vs CTRL	24 vs 38	NA	40 vs 39	8.4 vs 0.7	Simoa HD-1	22 vs 5	↑ in PA+A	33
		Plasma	PA vs A vs CTRL	3 vs 10 vs 8	67 vs 50 vs 63	38 vs 40 vs 48	0 vs 0 vs 11.5	Simoa HD-1	12 vs 22 vs 8	↑ in A > PA > CTRL<br>Associated with severity	39
		CSF	PA vs A vs CTRL	3 vs 7 vs 8	67 vs NA vs 63	38 vs 40 vs 48	0 vs 0 vs 11.5	Simoa HD-1	2412 vs 2615 vs 415	↑ A > CTRL	39
	Longitudinal	Plasma	PA+A vs CTRL	13 vs 19	NA	46 vs 50	10.2 vs 1.05	Simoa HD-1	26 vs 10	↑ in PA+A<br>Cut-off to A= 16.67 pg/mL<br>No changes after 2 yr	32
		Plasma	PA+A vs CTRL	9+6 vs 10	53 vs 40	38 vs 40	2 vs 0	Simoa HD-1	16 vs 5	↑ in PA+A<br>No changes after 1 yr	38
**SCA27B**	Cross-sectional	Plasma	SCA27B typical vs atypical [3] vs SCA3 vs MSA-C vs CTRL	6 vs 5 vs 30 vs 15 vs 15	NA	NA	NA	Simoa HD-X	22 vs 42 vs 30 vs 38 vs 11	↑ in atypical SCA27B ≈ SCA3 ≈ MSA-C > typical and CTRL	44
		Plasma	A vs CTRL	13 vs 38	NA	70 vs 39	11.3 vs 0.7	Simoa HD-X	17 vs 5	↑ in A	33
		Serum	SCA27B vs MSA-C vs SA vs CTRL	12 vs 26 vs 34 vs 19	NA	67 vs 66 vs 67 vs 63	NA	Simoa HD-1	16 vs 30 vs 16 vs 14	≈ in SA ≈ CTR; ↓ in MSA-C	46

Disease	Type of study	Sample	Groups	N	Sex (female, %)	Age (years)	SARA score	Assay/ Platform	NfL (pg/mL)	Main findings	Ref
**SCAs** [1]	Cross-sectional	Serum	SCAs vs CTRL	20 vs 20	50 vs 55	47 vs 46	1.0 vs NA	Simoa HD-1	59 vs 20	↑ in MSA-C > sporadic ataxia ≈ CTRL	23
**SCAs** [2]**/ MSA-C**	Cross-sectional	Plasma	SCAs vs MSA-C vs CTRL	60 vs 56 vs 74	50 vs 39 vs 55	50 vs 57 vs 55	10.6 vs 11.3 vs NA	Simoa HD-X	33 vs 42 vs 11	↑ in SCAs ≈ MSA-C > CTRL	41
**MSA-C**	Cross-sectional	Plasma	MSA-C+MSA-P vs CTRL	75+92 vs 40	44+56 vs 50	58+59 vs NA	NA	Simoa HD-1	40 vs 9	↑ in MSA;<br>Correlated with severity<br>MSA-C ≈ MSA-P	60
		Plasma	MSA-C vs SCAs vs CTRL	58 vs 29 vs 100	43 vs 45 vs 56	59 vs 47 vs 60	NA	Simoa HD-X	43 vs 34 vs 14	↑ in MSA-C > CTRL and SCAs	61
		Serum	MSA-C vs SA vs CTRL	25 and 25 vs 25	52 vs 52 vs 52	65 vs 59 vs 66	17.3 vs 11.3 vs NA	Simoa HD-1	61 vs 28 vs 33	↑ in MSA-C > SA and CTRL	23
		CSF	MSA-C+MSA-P vs CTRL	50+55 vs 40	44+56 vs 50	58+59 vs NA	NA	Simoa HD-1	4329 vs 560	↑ in MSA;	60
	Longitudinal	Plasma	MSA-C vs SAOA	33 and 65	44 vs 42	62 vs 65	14.5 vs 11.5	Simoa HD-1	29 vs 17	↑ in MSA-C > SLOA<br>↓ after disease onset	59
		Plasma	MSA-C vs MSA-P	15 vs 26	40 vs 58	62 vs 64	NA	Simoa HD-X	31 vs 44	↑ NfL in MSA-P > MSA-C<br>Correlates with severity<br>No changes after 1 year	62
		Plasma	MSA-C converter vs non-converter[4]	30 vs 42	43 vs 60	57 vs 55	NA	Simoa HD-X	30 vs 27	No differences	58
**MSA**	Cross-sectional	Serum	MSA vs CTRL	91 vs 60	48 vs 52	59 vs 59	NA	Simoa HD-X	33 vs 12	↑ in MSA.	63
	Longitudinal	Serum	MSA	64	NA	NA	NA	Simoa HD-X	32	↑ after 1 yr	63
**FRDA**	Cross-sectional	Plasma	A vs CTRL	33 vs 13	61 vs 54	34 vs 37	20.4 vs NA	Simoa HD-1	17 vs 8	↑ in A	49
		Plasma	A vs. CTRL	33 vs 12	44 vs 47	31 vs 32	22.6 vs NA	ELISA	18 vs 6	↑ in A	50
		Plasma	A	5	NA	43	25.4	Simoa	13	↑ in A	51
	Longitudinal	Plasma	A vs CTRL	12 vs 12	50 vs 50	15 vs 15	16.4 vs NA	ELLA	29 vs 5	↑ in A<br>No changes after 1 yr	48
		Plasma	A vs CTRL	50 vs 18	48 vs 50	28 vs 26	20.8 vs NA	ELLA	22 vs 6	↑ in A<br>No changes after 1 yr	48
		Plasma	A+PA vs CTRL	187 vs 127	53 vs 53	31 vs NA	NA	Simoa HD-1	NA	↑ in A+PA.<br>↓ with time in <35yr old	53
		Serum	PA vs A vs CTRL	13 vs 85 vs 19	46 vs 49 vs 47	38 vs 31 vs 36	NA	Simoa HD-1	8 vs 18 vs 8	↑ in A > PA ≈ CTRL<br>No changes after 1 yr	52
		Serum	A vs CTRL	99 vs 30	NA	38 vs 45	NA	Simoa HD-1	26 vs 21	↑ in A<br>No changes after 2 yr	54

Disease	Type of study	Sample	Groups	N	Sex (female, %)	Age (years)	SARA score	Assay/ Platform	NfL (pg/mL)	Main findings	Ref
**CANVAS**	Cross-sectional	Serum	CANVAS vs CTRL	61 vs 48	46 vs 44	67 vs 64	NA	Simoa HD-X	24 vs 12	↑ in CANVAS	56
**SPG7**	Cross-sectional	Plasma	A vs CTRL	17 vs 38	NA	58 vs 39	14.8 vs 0.7	Simoa HD-1	10 vs 5	No differences	33

Legend. Data are presented as mean or median. Values were rounded to the nearest integer (age, sex and NfL) or to one decimal place (SARA score). For longitudinal studies, the data presented correspond to baseline. Demographic and clinical data (age, sex and SARA) are reported as provided in each manuscript; due to missing data, in many studies the number of individuals characterized for age and/or SARA does not always correspond to the number of individuals included in the NfL analysis. **A**: Ataxic. **CTRL**: Control. **MSA**: Multiple System Atrophy. **MSA-C**: Multiple System Atrophy - Cerebellar phenotype. **MSA-P**: Multiple System Atrophy - Parkinson's phenotype. **NA**: Not Available. **PA:** Preataxic. **A**: Ataxic. **SA**: Sporadic Ataxia. **SAOA:** Sporadic Adult-Onset Ataxia. **SARA**: Scale for the Assessment and Rating of Ataxia. **Yr**: year/s [1] SCA1(n=6), SCA2 (n=3), SCA3 (n=8), SCA6 (n=3); [2] SCA1(n=2), SCA2 (n=7), SCA3 (n=27), SCA6 (n=1), SCA7 (n=1), SCA8 (n=4), SCA36 (n=1), other SCAs (n=17); [3] SCA27B typical: late onset (> 30 years old); SCA27B atypical: late onset (> 30 years old). [4] MSA-C converter: idiopathic late onset ataxia converted to MSA-C; MSA-C non-converter: idiopathic late onset ataxia non-converted to MSA-C.
